# Agreement and systematic bias between QuantiFERON chemiluminescent immunoassay and QuantiFERON enzyme-linked immunosorbent assay in the detection of latent tuberculosis infection: A systematic review and meta-analysis

**DOI:** 10.1016/j.ijregi.2025.100824

**Published:** 2025-12-07

**Authors:** Felix Bongomin, Ivaan Pitua, Phillip Ssekamatte, Diana Sitenda, Irene Andia-Biraro, Bwambale Jonani

**Affiliations:** 1 Department of Medical Microbiology and Immunology, Faculty of Medicine, Gulu University, Gulu, Uganda; 2 Department of Internal Medicine, School of Medicine, College of Health Sciences, Makerere University, Kampala, Uganda; 3 Tuberculosis and Comorbidities Research Consortium, Kampala, Uganda; 4 Department of Immunology and Molecular Biology, School of Biomedical Sciences, College of Health Sciences, Makerere University, Kampala, Uganda; 5 Department of Clinical Laboratory, Sebbi Hospital, Wakiso District, Uganda

**Keywords:** Latent tuberculosis, QuantiFERON, CLIA, ELISA, Method comparison, Agreement analysis

## Abstract

Objectives: Enzyme-linked immunosorbent assay (ELISA)–based QuantiFERON-TB Gold Plus (QFT-Plus ELISA) testing is associated with manual variability and indeterminate results. The transition from QFT-Plus ELISA to chemiluminescent immunoassay (CLIA) for QuantiFERON-TB Gold Plus (QFT-Plus CLIA) testing represents a significant methodological evolution that requires comprehensive evaluation. This systematic review and meta-analysis aimed to assess method agreement, characterize systematic bias patterns, evaluate clinical concordance between DiaSorin's QFT-Plus CLIA and QIAGEN's (QFT-Plus ELISA) methodologies, and determine sources of heterogeneity across diverse populations for latent tuberculosis infection (LTBI) detection.

Methods: We searched the PubMed, Embase, and Google Scholar databases from January 2019 to January 2025 for studies comparing QFT-Plus CLIA and QFT-Plus ELISA methodologies for LTBI detection. Two reviewers independently screened the studies and assessed their quality using the Quality Assessment of Diagnostic Accuracy Studies 2 (QUADAS-2) tool. We conducted a meta-analysis of agreement measures using random-effects models, systematic bias characterization, meta-regression analysis of the sources of heterogeneity, and clinical subgroup analysis. Evidence certainty was assessed using the Grading of Recommendations, Assessment, Development and Evaluation (GRADE). The review was registered with PROSPERO (CRD420251031884).

Results: A total of 16 studies comprising 4169 participants were included. A meta-analysis of 10 studies (n = 2932) revealed a substantial overall concordance of 88.76% (95% confidence interval [CI] 83.57-93.95). Among the seven studies with complete data to calculate positive and negative agreement measures (n = 1305), the pooled positive agreement was 91.3% (95% CI 88.95-93.65) and the negative agreement was 93.89% (95% CI 92.18-95.6). The comparative analysis revealed a mean discordance rate of 6.56% (range: 0.44-16.67%), with a weighted mean directional bias of +0.2% (showing minimal overall directional preference between platforms). Significant heterogeneity was observed (I² = 95.8%, *P* <0.0001), and evidence certainty was rated “very low” due to methodological limitations.

Conclusions: Diasorin's QFT-Plus CLIA and QIAGEN's QFT-Plus ELISA demonstrated substantial overall agreement in detecting LTBI, with the strongest concordance for clearly positive and negative results. Laboratories implementing QFT-Plus CLIA platforms should consider verification studies, and future research should evaluate the clinical significance of discordant results and optimize interpretive algorithms for diverse epidemiologic settings.

## Highlights

• QuantiFERON-TB chemiluminescent immunoassay and QuantiFERON-TB enzyme-linked immunosorbent assay showed an 88.76% overall agreement for latent tuberculosis infection detection.
• Platform discordance rate of 6.56% varied substantially across risk populations.
• High-risk groups demonstrated 8% lower agreement than low-risk populations.
• Systematic bias patterns ranged from −8.10% to +16.67% across diverse settings.
• Analytical agreement does not establish clinical equivalence between platforms.

## Introduction

Despite decades of control efforts, tuberculosis (TB) remains a formidable global health challenge. Recent World Health Organization (WHO) data indicate approximately 10.7 million people (95% uncertainty interval [UI]: 9.9-11.5 million) fell ill with TB in 2024, representing an incidence rate of 134 cases per 100,000 individuals [1]. With 1.23 million deaths attributed to TB (1.08 million deaths among HIV-negative people (95% UI: 0.99-1.18 million) and an estimated 150,000 deaths among people with HIV [95% UI: 120,000-183,000]), it has surpassed COVID-19 as the leading infectious cause of mortality worldwide [1].

Latent TB infection (LTBI) affects approximately one-fourth of the world’s population, with approximately two billion individuals harboring dormant bacilli with the potential for future reactivation [2]. Although most immunocompetent individuals with LTBI face only a 5-10% lifetime risk of progression to active disease, this risk increases dramatically among immunocompromised individuals. Therefore, the identification and treatment of LTBI in high-risk populations are the cornerstones of TB elimination strategies.

The traditional tuberculin skin test has several limitations, including cross-reactivity with Bacillus Calmette-Guerin vaccination and subjective interpretation [3]. The development of interferon-γ release assays has marked significant advancement in LTBI diagnosis, measuring T-cell responses to *Mycobacteria tuberculosis*–specific antigens absent from Bacillus Calmette-Guerin and most *non-tuberculous Mycobacteria*. QuantiFERON-TB Gold Plus (QFT-Plus) represents notable refinement of this technology, incorporating antigens that stimulate clusters of differentiations CD4+ and CD8+ T-lymphocytes to enhance detection sensitivity [4,5]. Initially developed for enzyme-linked immunosorbent assay (ELISA)–based platforms, QuantiFERON-TB assays have undergone significant technological evolution with the introduction of automated chemiluminescence platforms.

The LIAISON chemiluminescent immunoassay (QFT-Plus CLIA), recently endorsed by the WHO [6], uses QFT-Plus blood collection tubes, offering excellent sensitivity and specificity, greater automation potential, and significantly shorter turnaround times. QFT-Plus CLIA platform achieves faster turnaround times through a fully automated workflow using “flash” chemiluminescence technology with paramagnetic microparticles, enabling rapid signal generation and detection. The system can process up to 171 tests per hour, making it ideal for high-throughput applications.

The transition to QFT-Plus CLIA platform has raised several implementation questions: (i) neither methodology serves as the true gold standard for latent infection, (ii) method agreement rather than conventional diagnostic accuracy measures must guide decision-making, and (iii) the clinical impact of discordant results may vary across patient populations.

Several recent reviews have evaluated the diagnostic performance of interferon-γ release assays, including QIAreach QuantiFERON-TB, QFT-Plus ELISA, QuantiFERON-TB Gold In-Tube, and T-SPOT [7,8]. Among these, a meta-analysis by Mahmoudi and Hosseini Sharif [9] specifically assessed the diagnostic accuracy of QFT-Plus CLIA compared with the traditional QFT-Plus ELISA platform, reporting high sensitivity (0.97), high specificity (0.99), and strong overall agreement (0.92). However, that analysis evaluated QFT-Plus CLIA as an index test against a reference standard (QFT-Plus ELISA), which is a fundamentally different research question than the method agreement analysis between platforms.

When evaluating diagnostic tests, sensitivity and specificity metrics assess a platforms ability to identify diseases relative to the gold standard. Mahmoudi and Hosseini Sharif [9] used this diagnostic accuracy framework, demonstrating that QFT-CLIA performs reliably well for TB infection detection. However, this approach assumes that one methodology serves as the reference standard, an assumption that does not apply to LTBI detection where neither QFT-Plus ELISA nor QFT-Plus CLIA can directly detect dormant bacilli.

In the absence of a definitive reference standard, method comparison studies require additional analytical frameworks that focus on agreement metrics and systematic bias characterization [[10], [11], [12]]. Our meta-analysis addresses this distinct implementation challenge by quantifying the overall, positive, and negative agreement between QFT-Plus CLIA and QFT-Plus ELISA, systematic directional bias patterns, discordance rates across diverse populations, sources of heterogeneity through meta-regression, and population-specific variations through subgroup analysis.

These complementary approaches contribute to the understanding of whether laboratories can confidently transition between platforms, what systematic biases to expect, and how population characteristics modify the concordance. This distinction provides actionable guidance for laboratories globally implementing WHO-endorsed QFT-Plus CLIA technology [6], a contribution that is distinct from, yet complementary to the existing diagnostic accuracy literature.

## Materials and methods

### Literature search strategy

We conducted a systematic literature search following the Preferred Reporting Items for Systematic Reviews and Meta-Analyses (PRISMA) guidelines. We searched the PubMed, Embase, and Google Scholar databases for relevant publications from January 2019 to January 2025, with the final search performed on January 2, 2025. The search was structured according to the Population, Intervention, Comparison, Outcome framework and focused on human participants with suspected or confirmed LTBI. The intervention was the DiaSorin's QFT-Plus CLIA assay (QFT-CLIA) and the comparator was the QIAGEN's QFT-Plus ELISA. The outcomes of interest included agreement measures. The search terms included “(LIAISON) AND (TB),” “(TB Gold Plus) AND (LIAISON),” and “(QuantiFERON-TB Gold Plus) AND (LIAISON),” with filters for human studies without English language restrictions.

### Study selection criteria

We included peer-reviewed articles demonstrating QFT-Plus CLIA performance characteristics, clinical studies using human subject samples, comparative studies comparing device safety and performance, clinical or laboratory practice guidelines, and case reports of target conditions with low prevalence. We excluded studies that were not specific to device safety, performance, or diagnostic methodology; studies that did not use devices as intended; studies that combined results across multiple devices; studies that did not report safety or performance data; non-clinical performance studies; non-peer-reviewed sources; and duplicate manuscript records (early online and final published versions of the same study sharing identical data sets and Digital Object Identifiers).

The screening consisted of two levels: title and abstract review, followed by a full-text review. Data extraction used standardized forms, including first author, publication year, country, study design, sample size, population characteristics, cut-off values, and performance metrics, including positive agreement, negative agreement, overall agreement, κ statistics, and other performance metrics.

### Risk of bias assessment

The methodological quality was assessed using the Quality Assessment of Diagnostic Accuracy Studies 2 (QUADAS-2) tool, which evaluates four domains: patient selection, index test, reference standard, and flow and timing, along with three applicability domains. Each domain was rated as having a low, high, or unclear risk of bias based on predefined criteria tailored for diagnostic method comparison studies. We applied a predefined three-level framework to categorize the overall risk of bias (low, moderate, or high). Studies were classified as low risk if all four risk of bias domains were rated as low. Studies were rated as high risk if two or more domains were high or if one high-risk domain was accompanied by additional unclear ratings. All remaining studies, with one or more unclear domains but no serious flaws, were rated as having a moderate risk. Applicability concerns were reported separately and not included in the overall judgment.

### Statistical analysis

Data analysis was performed using R software (version 4.5.1). For studies with sufficient data, 2 × 2 contingency tables were constructed. Positive agreement was calculated as the percentage of samples positive by both methods among all samples positive by either method. Negative agreement was calculated as the percentage of samples negative by both methods among all samples negative by either method. We conducted bias characterization using platform-specific bias patterns and discordance rates. A meta-analysis was conducted examining heterogeneity sources using sample size, publication year, geographic region, and population risk factors as covariates.A clinical subgroup analysis was conducted by stratifying agreement by risk populations and correlation analysis was used to assess the relationship between positive and negative agreement patterns. Heterogeneity was evaluated using the I² statistic.

## Results

### Search results

**Figure 1:**
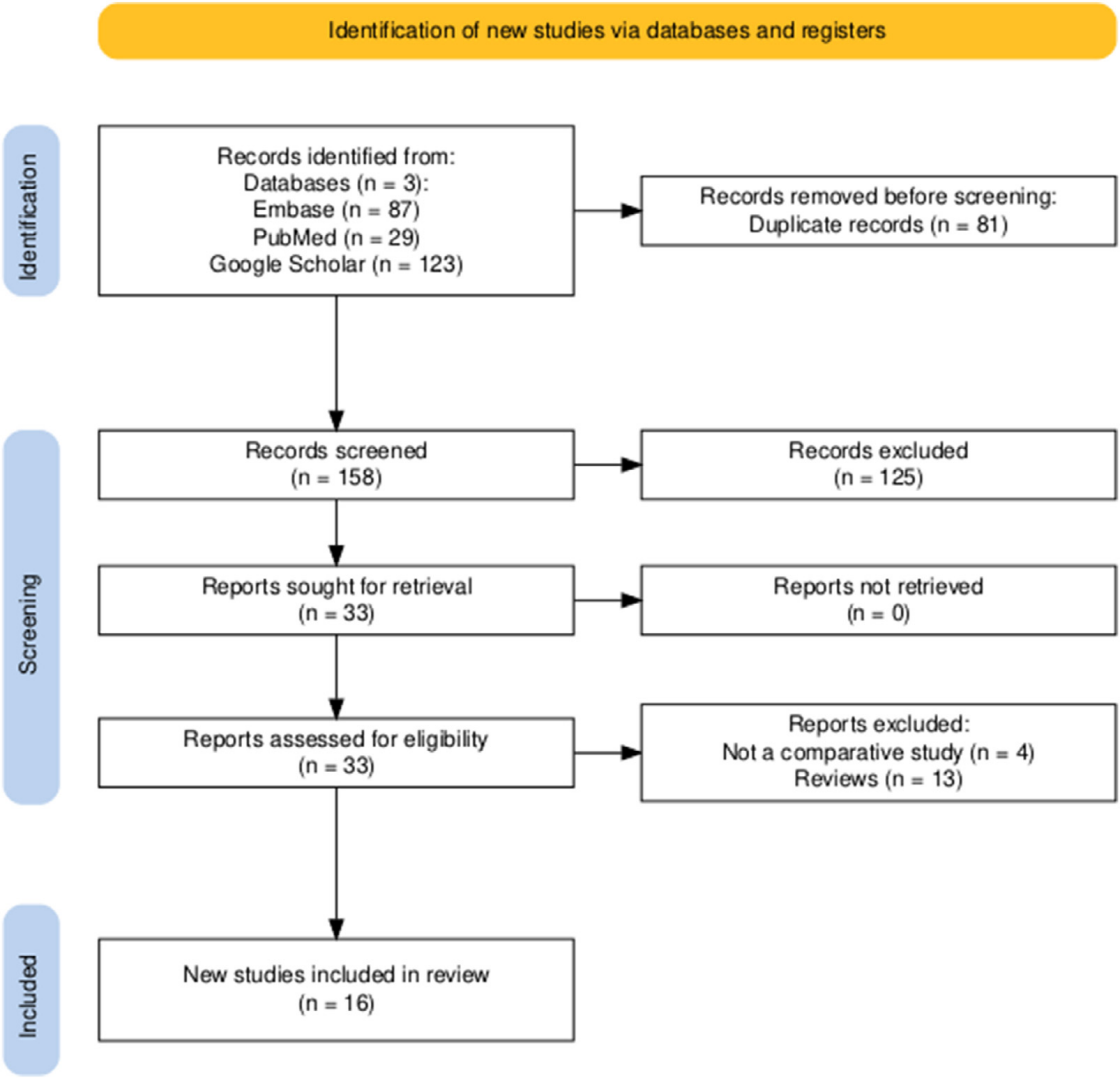
Literature search flow chart (PRISMA).

The search yielded 239 records. After removing duplicates, 157 records were retained. An independent review by two reviewers resulted in 32 eligible studies (S1 Table). After a detailed full-text review and quality appraisal, 16 studies [[13], [14], [15], [16], [17], [18], [19], [20], [21], [22], [23], [24], [25], [26], [27], [28]] were included in the final analysis (Figure 1).

### Study characteristics

A total of 16 studies from diverse countries (United States, Italy, Belgium, Sweden, Spain, and Chile) predominantly used convenience sampling to evaluate QFT-Plus CLIA vs QFT-Plus ELISA in routine clinical populations including TB contacts, health care workers, and hospitalized patients. The studies were published between 2019 and 2024, with sample sizes ranging from 36 to 807 participants (median 176) (Table 1). All studies compared QFT-Plus CLIA with the conventional QFT-Plus ELISA method using the manufacturer-recommended cut-off value of 0.35 IU/ml for both methods.

**Table 1:** Study characteristics including author, year, country, study design, sample size, and population.

First author *et al.*	Year	Country	Study design	Sample size	Sampling method	Population/sample description	Age range (years)
Buron and Banaei [19]	2023	USA	Comparative study	278	Convenience sampling	Adult patients tested routinely for latent tuberculosis infection; 18.9% infected with SARS-CoV-2	42-81
Bisognin et al. [13]	2020	Italy	Comparative study	419	Convenience sampling	Household contacts (≥6 years old) of active pulmonary TB cases, mostly BCG-vaccinated	≥6 years
Grassi et al. [21]	2019	Italy	Comparative study	807	Purposive sampling	Routine patient samples for *M. tuberculosis* testing	Not specified
Mehreen et al. [28]	2021	USA	Comparative Study	40	Convenience sampling	Specimens with antigen-nil results between 0.35 and 1.00 IU/ml using QFT-Plus CLIA	Not specified
Khoury et al. [14]	2023	USA	Comparative study	452	Convenience sampling	Samples collected from long-term care facilities resident were run over 9 months	Not specified
Brantestig et al. [15]	2020	Sweden	Comparative study	125	Purposive sampling	Specimens submitted to Karolinska University Hospital for QuantiFERON-TB Gold Plus analysis	
Heireman et al. [16]	2022	Belgium	Comparative study	125	Convenience sampling	Patients from low–tuberculosis incidence setting (Belgium). Six patients had been diagnosed with active tuberculosis (polymerase chain reaction–confirmed).	Median 58 years (Range 5-89)
de Maertelaere et al. [17]	2020	Belgium	Cross sectional study	92	Convenience sampling	Heparin blood samples taken for routine QFT assays. Four samples were from encoded health care workers sent by the occupational medicine department	1 to 91
Fernández-Huerta et al. [20]	2021	Spain	Comparative study	333	Convenience sampling	Samples collected for the routine performance of the QFT-Plus test at the Bellvitge University Hospital. 61 study patients (18.9%) were infected with SARS-CoV-2	Not specified
Villalta et al. [18]	2020	Italy	Comparative study	157	Convenience sampling	Samples taken from routine testing from females and males of median age 51 years	5-81
Altawallbeh et al. [24]	2021	USA	Cross sectional study	329	Purposive sampling	Three cohorts, including 15 with confirmed TB (active TB cohort), 129 non-TB (low risk cohort), and 185 potential TB (mixed risk cohort).	18-74 years
Alessio et al. [23]	2022	Italy	Observational study	36	Purposive sampling	Patients hospitalized because of COVID-19 who had received an indeterminate QFT-Plus ELISA test during hospitalization	Median 58 years
Cornaby et al. [26]	2022	USA	Observational study	481	Convenience sampling	Subjects included outpatients and inpatients presenting to the UNC Health Care system from April 2020 to August 2021 with a positive	Not specified
Kadkhoda et al. [25]	2023	USA	Comparative study	190	Random sampling	Randomly-selected, de-identified residual plasma samples with known initial negative, indeterminate, and positive results	Not specified
Ruiz-Tagle et al. [22]	2024	Chile	Prospective cohort study	144	Consecutive sampling from three health service areas	A prospective cohort of TB household contacts	Not specified
Stojkovic et al. [27]	2019	Belgium	Comparative study	161	Not specified	Samples for routine TB testing	Not specified

TB, tuberculosis; QFT, QuantiFERON®-TB Gold; QFT-Plus, QuantiFERON®-TB Gold Plus.

### Risk of bias

Most studies demonstrated generally good participant recruitment procedures. However, the reporting of index test procedures was limited, and 15 studies were rated as having at least one unclear element in this domain. Reference standard and flow/timing assessments showed greater variation (S2 Table).

A total of 10 studies [13,[16], [17], [18], [19], [20], [21],[24], [25], [26]] were judged to have a moderate risk of bias, typically due to incomplete reporting in one or more domains without evidence of major methodological flaws. Five studies [14,15,23,27,28] demonstrated a high risk of bias (S2 Table).

### Overall agreement

**Figure 2:**
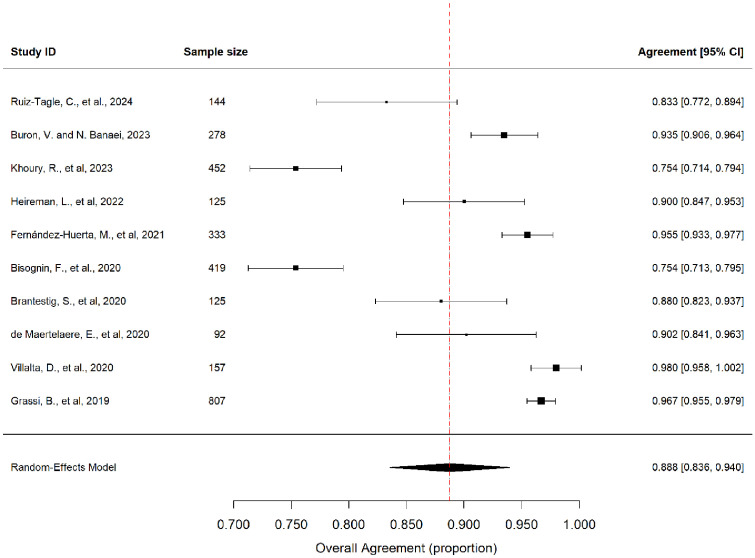
Forest plot of overall agreement estimates across the included studies. Pooled overall agreement: 88.76% (95% CI: 83.57-93.95%); I² = 96.7%, P <0.0001.

A meta-analysis of 10 studies (n = 2932) that reported overall agreement [[13], [14], [15], [16], [17], [18], [19], [20], [21], [22]] found an overall pooled agreement of 88.76% (95% confidence interval [CI]: 83.57-93.95%) using a random-effects model with inverse-variance weighting, although substantial heterogeneity was observed (I² = 96.7%, *P* <0.0001) (Figure 2). Cohen κ values, reported in five studies, ranged from 0.61 to 0.88, indicating substantial to almost perfect agreement beyond chance.

CI, confidence interval.

### Positive and negative agreement between QFT-Plus CLIA and QFT-Plus ELISA

Seven studies [[13], [14], [15], [16],^18^,^19^,^22^] provided complete data for constructing 2 × 2 contingency tables and calculating agreement measures (Table 2). The pooled positive agreement between QFT-Plus CLIA and QFT-Plus ELISA was 91.30% (95% CI: 88.95-93.65%), and the negative agreement was 93.89% (95% CI: 92.18-95.60%).

**Table 2:** Positive and negative agreement between QFT-CLIA and QFT-ELISA assays.

Study	Year	QFT-Plus CLIA+/QFT-Plus ELISA+	QFT-Plus CLIA+/QFT-Plus ELISA–	QFT-Plus CLIA–/QFT-Plus ELISA–	QFT-Plus CLIA–/QFT-Plus ELISA+	Positive agreement (%)	Negative agreement (%)
Buron and Banaei [19]	2023	121	11	139	7	91.67	95.21
Bisognin et al. [13]	2020	104	0	122	1	100.00	99.19
Khoury et al. [14]	2023	146	7	135	33	95.42	80.36
Brantestig et al. [15]	2020	60	0	51	3	100.00	94.44
Heireman et al. [16]	2022	19	3	93	1	86.36	98.94
Villalta et al. [18]	2020	11	3	90	1	78.57	98.90
Ruiz-Tagle et al. [22]	2024	43	24	77	0	64.18	100.00
Pooled		504	48	707	46	91.30	93.89

QFT-Plus CLIA, DiaSorin's QuantiFERON-TB Gold Plus; QFT-Plus ELISA, QIAGEN's QuantiFERON-TB enzyme-linked immunosorbent assay.

### Systematic bias analysis

**Figure 3:**
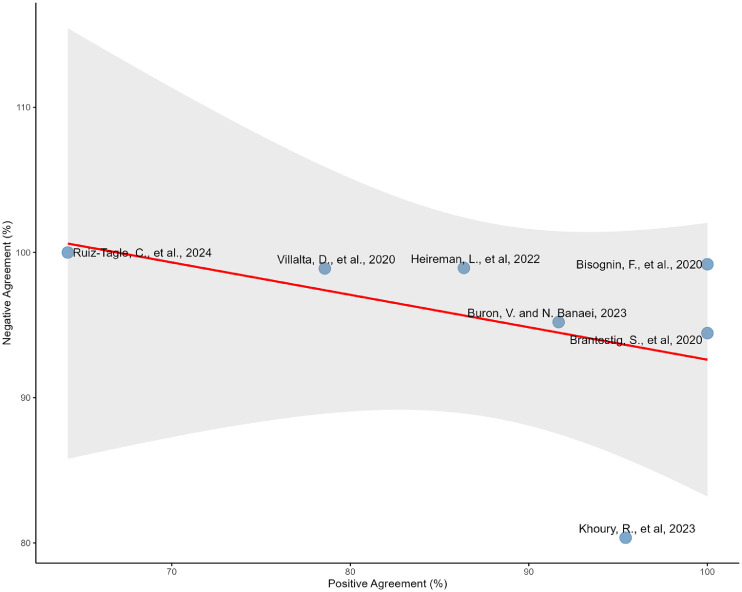
Scatter plot showing the relationship between positive and negative agreement measures across seven studies comparing DiaSorin's QuantiFERON-TB Gold Plus and QIAGEN's QuantiFERON-TB enzyme-linked immunosorbent assay testing platforms. Pearson correlation r = −0.4197 (P = 0.348), indicating non significant correlation between positive and negative agreement patterns.

Comparative analysis across the seven studies (n = 1305) [[13], [14], [15], [16],^18^,^19^,^22^] revealed a mean discordance rate of 6.56% (range: 0.44-16.67%). Overall, the directional bias analysis showed minimal systematic differences between QFT-Plus CLIA and QFT-Plus ELISA, with a weighted mean bias of +0.2%. However, the bias direction varied substantially across studies, ranging from −8.10% to +16.67%, underscoring the importance of contextual interpretation near the diagnostic cutoff. The correlation between positive and negative agreement was −0.4197 (*P* = 0.348) (Figure 3).

### Clinical subgroup analysis

Subgroup analysis revealed differential agreement patterns across the risk populations. High-risk populations showed lower overall agreement (83.0%, 95% CI: 74.6-91.3%) than low-risk populations (91.0%, 95% CI: 85.3-96.8%). Large studies (≥300 participants) demonstrated lower agreement (85.8%) than smaller studies (90.5%), suggesting potential volume-dependent effects on the precision of agreement.

### Effect of sample size on precision of measurement

A significant negative correlation (Spearman ρ = −0.705, *P* = 0.023) was observed between sample size and measurement precision (standard error), indicating that larger studies provided more reliable agreement estimates with narrower CIs.

### Publication bias

Visual inspection of the funnel plot revealed asymmetry (S1 Figure). Despite this asymmetry, the leave-one-out sensitivity analysis demonstrated remarkable consistency in pooled estimates ranging from 87.68% to 90.32%, with overlapping CIs.

### Method comparison characteristics

After the diagnostic platform transition, operational data revealed an increased frequency of low-positive results with the QFT-Plus CLIA assay (1.3-5.4%), exceeding the expected baseline of 2-3%. Mehreen *et al.* [28] found that among 40 retested samples, 70% returned positive results with the QFT-Plus CLIA assay but were negative with QFT-Plus ELISA. When they performed clinical reviews of patient histories, they did not yield consistent explanatory factors, suggesting that the observed discordance reflected inherent platform differences rather than patient-specific factors.

### Heterogeneity exploration

The meta-regression analysis revealed no significant association between the overall agreement and sample size (coefficient = 0.0000, *P* = 0.9727), publication year (coefficient = −0.0177, *P* = 0.2806), or population risk level (coefficient = −0.0844, *P* = 0.1193). The observed substantial heterogeneity (I² = 96.7%) appears to stem from methodological rather than demographic factors.

### Narrative synthesis: beyond concordance statistics

The narrative synthesis of five studies [[23], [24], [25], [26], [27]] emphasized three critical implementation themes that added essential context for clinical and laboratory decision-making.

### Population risk stratification emerges as a determinant of platform performance

Evidence has demonstrated that platform agreement varies systematically across risk populations, with high-risk groups showing consistently lower concordance rates than low-risk populations. Altawallbeh *et al.* [24] found that patients with active TB achieved only 92.8% agreement (κ = 0.76) compared with 97.9% agreement (κ = 0.74) in low-risk populations. This risk-dependent performance pattern suggests that the epidemiologic context fundamentally influences platform reliability. Among immunocompromised populations, particularly, patients with COVID-19 with indeterminate results, platform discordance becomes amplified, with mitogen responses differing markedly between QFT-Plus CLIA and QFT-Plus ELISA methodologies (10.0 vs 0.3 IU/ml, *P* <0.0001) [23]. These observations indicate that simple concordance statistics may inadequately capture platform performance in clinically vulnerable populations where diagnostic accuracy has heightened consequences.

### Reproducibility limitations challenge clinical interpretation

In addition to inter-platform agreement, intra-platform reproducibility is a significant concern for QFT-Plus CLIA methodology. Cornaby *et al.* [26] found that repeat testing of initially positive samples demonstrated substantial variability, with only 73-74% concordance for individual TB antigens upon retesting. This reproducibility deficit necessitates threshold-based interpretation strategies, where samples exceeding specific cut-off values (≥4.54 IU/ml for TB 1 and ≥4.78 IU/ml for TB 2) achieve reliable repeatability. This suggests that borderline positive results may require confirmation testing, particularly, in low-prevalence settings, where false-positive results carry significant clinical and economic implications.

### Analytical performance validates technical equivalence

Despite variations in clinical concordance, analytical validation has demonstrated robust technical performance. Stojkovic *et al.* [27] reported excellent reproducibility (coefficient of variation 2.7-3.9%) and strong inter-platform correlation (r = 0.956), with systematic bias approaching zero near the diagnostic threshold (0.01 IU/ml at 0.35 IU/ml cutoff). Kadkhoda *et al.* [25] strengthened this evidence through methodologically rigorous random sampling with stratified selection across the result categories (negative, indeterminate, and positive). Thier findings showed over all acceptable coefficient of variations (CVs) that were less than 20%. Cutoff verification showed CVs for QFT-Plus ELISA TB1 and TB2 (5.2%, 4.1%), and QFT-Plus CLIA TB1 and TB2 (5.2%, 5.4%) respectively, demonstrating robustness around the cutoffs.

### Certainty of evidence

For positive and negative agreement, the evidence certainty was downgraded to low and very low, respectively, due to the serious risk of bias from inadequate blinding and selective reporting in six non-randomized studies (n = 1161). The overall agreement from nine studies (n = 2788) was downgraded to very low certainty due to a serious risk of bias and suspected publication bias, as evidenced by funnel plot asymmetry. (S1 Figure).

## Discussion

This systematic review and meta-analysis demonstrated substantial concordance between the QFT-Plus CLIA and QFT-Plus ELISA methodologies, 88.76% (95% CI: 83.57-93.95%) across 10 studies comprising 2932 participants. Although positive and negative agreements reached 91.30% and 93.89%, respectively, the analysis revealed variable agreement patterns and population-specific variations, with important implications for laboratory practice and clinical interpretation of results. Comparative analysis revealed a mean discordance rate of 6.56% (range: 0.44-16.67%) between the platforms. Directional bias analysis showed minimal overall systematic differences between platforms (weighted mean bias: +0.2%), although the bias direction varied considerably across studies (range: −8.10% to +16.67%). This substantial variation indicates that platform-specific effects are modified by population characteristics and laboratory conditions rather than representing consistent analytical differences favoring either platform.

Individual study analyses provide additional insights into platform differences. Buron and Banaei [19] reported through a Bland–Altman analysis that QFT-Plus CLIA consistently yielded higher interferon-γ concentrations than QFT-Plus ELISA (mean bias: 0.21 IU/ml), with this analytical difference concentrated near the diagnostic threshold (0.35IU/ml). This finding aligns with method comparison studies for other immunoassay platforms transitioning from ELISA to CLIA methodologies, which have reported a systematic bias toward higher readings with chemiluminescent technologies while maintaining a high overall agreement [29,30]. However, our meta-analytic findings demonstrate that higher analytical concentrations do not consistently translate into more positive classifications across all populations and laboratory settings.

Laboratories transitioning from QFT-Plus ELISA to QFT-Plus CLIA platforms should implement verification studies focusing on near-threshold samples (0.2-0.7 IU/ml range), where platform discordance is most pronounced and clinical decision-making is most affected. The observed discordance patterns suggest the implementation of transitional monitoring protocols after implementation, with particular attention to unexpected results in low-prevalence populations, where discordant findings have greater clinical consequences.

The transition from QFT-Plus ELISA to QFT-Plus CLIA platforms raises important questions regarding manufacturer responsibility and laboratory implementation strategies. Although the QFT-Plus CLIA assay has received a WHO endorsement for TB infection diagnosis [6], the primary responsibility for ensuring analytical equivalence and clinical interchangeability between platforms rests with the manufacturer rather than individual clinical laboratories. The observed discordance rate of 6.56% and substantial variability in directional bias (ranging from −8.10% to +16.67% across studies) suggest that platform-specific differences may have clinical implications, particularly, in borderline results near the 0.35 IU/ml diagnostic threshold level. The manufacturer’s recommendation for “repeat testing of unexpected positive results” [31] requires clearer operational definition because the term “unexpected” remains ambiguous without reference to clinical context, pre-test probability, or risk stratification. In practice, some laboratories have implemented confirmatory QFT-Plus ELISA testing for QFT-Plus CLIA results between 0.35 and 1.00 IU/mL to mitigate potential false-positive results. However, this approach is neither cost-effective nor sustainable on a large scale. Addtionally, the observation that QFT-Plus CLIA results tend to yield higher interferon-γ values than QFT-Plus ELISA in some contexts [19] could lead to increased rates of preventive therapy in individuals who may not be truly infected, with attendant costs and toxicity implications. These findings underscore the need for manufacturer-level harmonization of interpretive guidelines and development of evidence-based algorithms that account for result magnitude, population prevalence, and individual risk factors rather than relying solely on dichotomous cutoffs.

Our subgroup analysis revealed differential agreement patterns that varied significantly according to the population risk profile and geographic setting. High-risk populations demonstrated a notably lower overall agreement (83.0%, 95% CI: 74.6-91.3%) than low-risk populations (91.0%, 95% CI: 85.3-96.8%), representing an 8.0% difference with clear clinical implications. This variation suggests that clinical interpretation algorithms should incorporate epidemiologic risk factors when managing discordant results rather than relying solely on quantitative thresholds.

Platform discordance was most pronounced near the diagnostic threshold of 0.35 IU/ml, where the interpretive uncertainty is inherently the highest. Although some studies have proposed borderline interpretive zones for QuantiFERON assays, particularly, in the serial testing of health care workers, to account for variability near the diagnostic cutoff [32], our analysis did not support consistent retesting outcomes within this range. Consistent with current DiaSorin recommendations, repeat testing should be considered for unexpected positive results, particularly, in low-prevalence settings, with interpretation guided by a comprehensive TB risk assessment [31].

The significant negative correlation between sample size and measurement precision (Spearman ρ = −0.695, *P* = 0.038) indicates that larger studies provide more reliable agreement estimates, supporting the robustness of the findings from well-powered studies. This methodological observation has important implications for future study designs in platform comparison research.

The clinical utility of QFT-Plus CLIA testing depends critically on the appropriate interpretation of the results within the broader context of tuberculosis risk assessment. Laboratory transitions should integrate QFT-Plus CLIA results with clinical judgment, epidemiologic factors, and complementary laboratory findings rather than relying solely on quantitative thresholds. This approach helps resolve discordant findings and ensures that diagnostic decisions are informed by the assay performance characteristics and epidemiologic context.

### Study limitations and research implications

The current evidence base provides useful information on the analytical agreement between the QFT-Plus CLIA and QFT-Plus ELISA platforms but has important limitations in terms of scope and interpretation. The high analytical agreement does not necessarily establish clinical equivalence or interchangeability. Our analysis was limited to examining the concordance of categorical results and did not assess the clinical impact of discordant results, such as rates of unnecessary preventive therapy, cost-effectiveness of platform transitions, or differential outcomes in progression to active TB based on platform-specific results. These limitations constrain the utility of our findings for decision-making regarding platform replacement, underscoring why our conclusions should be interpreted as descriptive comparisons rather than definitive guidance for clinical interchangeability.

We acknowledge the substantial methodological heterogeneity across studies, absence of clinical outcome data correlating discordant results with progression to active TB, lack of standardized blinding protocols, and non-randomized sample selection bias among the studies included in this review. The “very low” certainty rating using the GRADE methodology reflects these methodological limitations and potential publication bias, as evidenced by funnel plot asymmetry.

These limitations highlight critical research gaps that should be addressed through longitudinal studies with randomized participant selection, standardized blinding of results from each testing platform, and systematic evaluation of clinical outcomes following the discordant results. Future research should prioritize determining whether platform-specific discordance translates into meaningful differences in clinical outcomes, particularly, the progression to active TB in diverse epidemiological settings.

## Conclusion

DiaSorin's QFT-Plus CLIA and QIAGEN's QFT-Plus ELISA demonstrat a substantial overall agreement in detecting LTBI (88.76%), with strongest concordance for clearly positive and negative results. However, the current evidence base, although informative for analytical comparison, does not provide sufficient data to definitively establish clinical interchangeability or recommend the unconditional replacement of QFT-Plus ELISA with QFT-Plus CLIA platforms. The observed discordance patterns concentrated near the diagnostic threshold (0.35 IU/ml), with substantial between-study heterogeneity, and very low certainty of evidence (GRADE assessment), which indicate that decisions regarding platform transition should be made cautiously and with appropriate clinical oversight.

## Declaration of competing interest

The authors have no competing interests to declare.

## Appendix Supplementary materials

Image, application 1

Image, application 2

Image, application 3

Image, application 4

Image, application 5
