# Supplementary figures and images for: Agreement and systematic bias between QuantiFERON chemiluminescent immunoassay and QuantiFERON enzyme-linked immunosorbent assay in the detection of latent tuberculosis infection: A systematic review and meta-analysis
Source: IJID Reg. 2025 Dec 7;18:100824. doi: 10.1016/j.ijregi.2025.100824 (PMC12809075; doi:10.1016/j.ijregi.2025.100824)

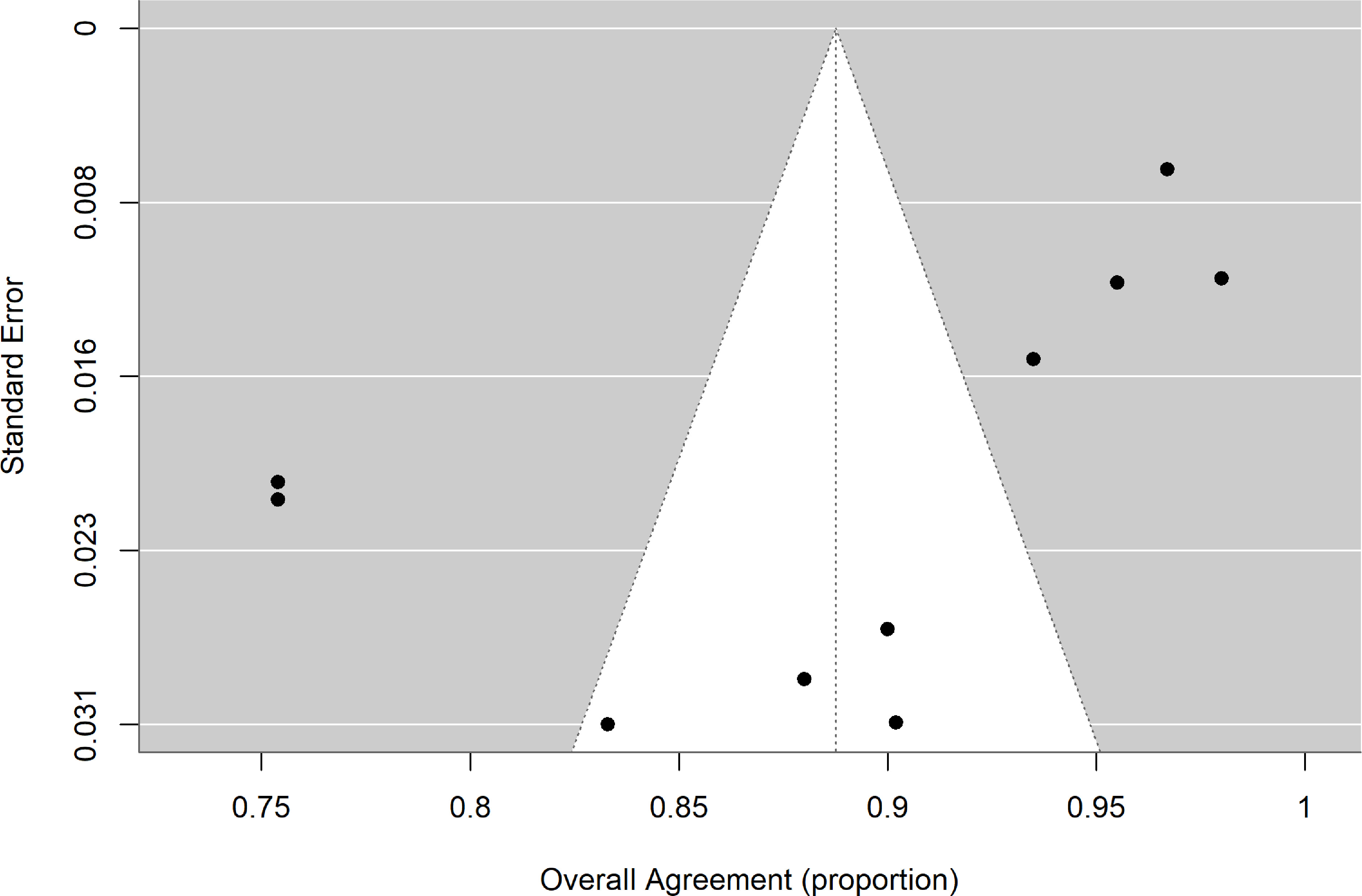

Supplement: Supplementary file 2 [file mmc2.jpg]
