# Supplementary material for: Agreement and systematic bias between QuantiFERON chemiluminescent immunoassay and QuantiFERON enzyme-linked immunosorbent assay in the detection of latent tuberculosis infection: A systematic review and meta-analysis
Source: IJID Reg. 2025 Dec 7;18:100824. doi: 10.1016/j.ijregi.2025.100824 (PMC12809075; doi:10.1016/j.ijregi.2025.100824)
Supplement: Supplementary file 3 [file mmc3.pdf]

S1 Table. Studies excluded for the systematic review and Meta-analysis

Serial No	Authors and Title	Reason for Exclusion
1.	Alonzi, T., et al. (2023). "Research tests for the diagnosis of tuberculosis infection." Expert Review of Molecular Diagnostics 23(9): 783-795.	This is a review. We can't get primary data from it
2.	Buron, V. and N. Banaei (2023)."Erratum: Inflated Gamma Interferon Response with QuantiFERON-TB Gold Plus Using the Automated Liaison XL Analyzer: a Testing Algorithm To Mitigate False-Positive Results in Low-Incidence Settings (Journal of Clinical Microbiology (2023) 61:6 (e00295-23) DOI: 10.1128/jcm.00295-23)." Journal of Clinical Microbiology 61(11).	Duplicated record
3.	Busà, R., et al. (2022). "Specific Anti-SARS-CoV-2 Humoral and Cellular Immune Responses After Booster Dose of BNT162b2 Pfizer-BioNTech mRNA-Based Vaccine: Integrated Study of Adaptive Immune System Components." Frontiers in Immunology 13.	*Does not report on the intended patient population*
4.	Chun, H. M., et al. (2022). "A Systematic Review of COVID-19 Vaccine Antibody Responses in People With HIV." Open Forum Infectious Diseases 9(11).	*Does not report on the intended patient population*
5.	Cleary, A., et al. (2020). "An Audit of Emergency Tuberculosis Admissions to a Tertiary Referral Hospital in Ireland." Irish Journal of Medical Science 189(SUPPL 3): S24.	*Uses an alternate device*
6.	Cleary, A., et al. (2019). "An audit of emergency tuberculosis admissions to a tertiary referral hospital in Ireland." Irish Journal of Medical Science 188: S315.	*Uses an alternate device*
7.	Cortez, R., et al. (2024). "Evaluation of the Use of One-tube Blood Collection and Automated Specimen Processing System for LIAISON® QuantiFERON®-TB Gold Plus Testing at a Large Regional Reference Laboratory." Clinical Chemistry 70: i96.	*Using samples not from human*
8.	Damen, L., et al. (2021). "Bone mineral density during 3 years of growth hormone in previously GH-treated young adults with PWS." European Journal of Endocrinology 184(6): 773-782.	*Does not report on the intended patient population*
9.	Desmecht, S., et al. (2022). "Kinetics and Persistence of the Cellular and Humoral Immune Responses to BNT162b2 mRNA Vaccine in SARS-CoV-2-Naive and -Experienced Subjects: Impact of Booster Dose and Breakthrough Infections." Frontiers in Immunology 13.	*Does not report on the intended patient population*
10.	Escalante, P., et al. (2023). "New diagnostics for the spectrum of asymptomatic TB: from infection to subclinical disease." International Journal of Tuberculosis and Lung Disease 27(7): 499-505.	Narrative review
11.	Fernández-Moreno, R., et al. (2024). "QuantiFERON–CMV assay by chemiluminescence immunoassay: Is it more suitable for real-live monitoring of transplant patients?" Journal of Clinical Virology 171.	*Uses an alternate device*
12.	Firoj, S. and N. Annapurna (2024). "A Study Of Psychiatric disorders In patients With Tuberculosis." Indian Journal of Psychiatry 66: S116-S117.	*Uses an alternate device*
13.	Gallini, N., et al. (2023). "Implementation and impact of the global access principles at the University of British Columbia: current successes and future challenges." Frontiers in Pharmacology 14.	*Does not report on the intended patient population*
14.	Geller, J. (2020). "US Food and Drug Administration Announces Availability of Important Final Guidance." Journal of Clinical Engineering 45(2): 103-109.	*Does not report on the intended patient population*
15.	Gong, W. and X. Wu (2021). "Differential Diagnosis of Latent Tuberculosis Infection and Active Tuberculosis: A Key to a Successful Tuberculosis Control Strategy." Frontiers in Microbiology 12.	Narrative review
16.	Gunawardena, S. M., et al. (2024). "A systematic review of the prevalence of psychosis in people with tuberculosis." European Psychiatry 67: S370.	*Uses an alternate device*
17.	Hamada, Y., et al. (2021). "Tests for tuberculosis infection: Landscape analysis." European Respiratory Journal 58(5).	Narrative review
18.	Jafri, S., et al. (2019). "LIAISON OF SUGAR CONTROL WITH TIME TO SPUTUM SMEAR CONVERSION IN MULTIDRUG-RESISTANT TB." Chest 156(4): A362.	*Uses an alternate device*
19.	Jaleel, A., et al. (2024). "Interplay of interleukins (IL6, IL10) and 25 hydroxycholecalciferol in asthmatic subjects with chronic post-COVID condition (PCC)." Tropical biomedicine 41(1): 70-77.	*Does not report on the intended patient population*

20.	Jayasooriya, S., et al. (2019). "The burden of non-TB lung disease presenting to TB clinics in the Gambia: Preliminary data in the xpert® MTB/rif era." Public Health Action 9(4): 166-168.	*Uses an alternate device*
21.	Kadkhoda, K., et al. (2022). Comparison of LIAISON QuantiFERON-TB Gold Plus with QuantiFERON-TB Gold Plus.	*Primarily contains duplicate data from another included study*
22.	Kobashi, Y. (2023). "Current status and future landscape of diagnosing tuberculosis infection." Respiratory Investigation 61(5): 563-578.	Narrative review
23.	Kobayashi, Y., et al. (2022). "Multidrug-Resistant Tuberculosis Outbreak among Immigrants in Tokyo, Japan, 2019–2021." Japanese Journal of Infectious Diseases 75(5): 527-529.	*Uses an alternate device*
24.	La Manna, M. P., et al. (2022). "Impact of Mycobacterium tuberculosis Infection on Human B Cell Compartment and Antibody Responses." Cells 11(18).	*Uses an alternate device*
25.	Labdon, C. (2019). "LIAISON QuantiFERON-TB gold plus: Automation in LTBI testing." Clinica Chimica Acta 493: S775.	No application of scientific method
26.	Lampaert, E., et al. (2024). "Evaluation of centralised and decentralised models of care during the 2020 Ebola Virus Disease outbreak in Equateur Province, Democratic Republic of the Congo: A brief report." F1000Research 13.	*Does not report on the intended patient population*
27.	Mafi, S., et al. (2023). "Evaluation of the fully automated LIAISON®XL chemiluminescence analyzer for QuantiFERON®-CMV testing in transplant recipients." Journal of Clinical Virology 166.	*Uses an alternate device*
28.	Marks, M. R., et al. (2022). "(49) Race and Gender Disparities in Antipsychotic Medications and Consultation-Liaison Psychiatry Involvement in a Trauma Center." Journal of the Academy of Consultation-Liaison Psychiatry 63: S136-S137.	*Does not report on the intended patient population*
29.	Masiá, M., et al. (2023). "Integrating SARS-CoV-2-specific interferon-γ release assay testing in the evaluation of patients hospitalized with COVID-19." Microbiology Spectrum 11(6).	*Does not report on the intended patient population*
30.	Mayorga Ayala, L. F., et al. (2022). "T cell response to SARS-CoV-2 mRNA vaccines by an interferon-gamma release immunoassay in patients with Inflammatory Bowel disease receiving anti-TNF and thiopurine treatment." Journal of Crohn's and Colitis 16: i525.	*Does not report on the intended patient population*
31.	Newson, T. P. and O. Smith (2020). "Childhood tuberculosis still a significant burden: Experience of a district dedicated tuberculosis service." Archives of Disease in Childhood 105(SUPPL 1): A211.	*Uses an alternate device*
32.	Niimi, R., et al. (2022). "Relationships between QUS and HR-pQCT, DXA, and bone turnover markers." Journal of Bone and Mineral Metabolism 40(5): 790-800.	*Does not report on the intended patient population*
33.	Park, I. U., et al. (2020). "Sensitivity and Specificity of Treponemal-specific Tests for the Diagnosis of Syphilis." Clinical Infectious Diseases 71: S13-S20.	*Does not report on the intended patient population*
34.	Pinto, A. C., et al. (2022). "EVALUATING A CLINIC FOR UNACCOMPANIED ASYLUMSEEKING CHILDREN (UASC): THE IMPORTANCE OF INFECTIOUS DISEASE SCREENING AND A HOLISTIC APPROACH TO CARE." Archives of Disease in Childhood 107: A327-A328.	*Uses an alternate device*
35.	Player, M. (2020). "A different kind of OOPE, volunteering to improve haematology services in Malawi." British Journal of Haematology 189: 65.	*Does not report on the intended patient population*
36.	Prestileo, T., et al. (2021). "Tuberculosis among migrant populations in sicily: A field report." Journal of Tropical Medicine 2021.	*Uses an alternate device*
37.	Ragonnet, R., et al. (2022). "Estimating the long-term effects of mass screening for latent and active tuberculosis in the Marshall Islands." International Journal of Epidemiology 51(5): 1433-1445.	*Uses an alternate device*
38.	Rakshit, S., et al. (2022). "Evidence for the heterologous benefits of prior BCG vaccination on COVISHIELD™ vaccine-induced immune responses in SARS-CoV-2 seronegative young Indian adults." Frontiers in Immunology 13.	*Does not report on the intended patient population*
39.	Ramasamy, R., et al. (2020). "Dynamic method for liaison of community pharmacists with national programme for tuberculosis control: Efforts to harness untapped opportunities." Indian Journal of Pharmaceutical Education and Research 54(3): 809-818.	*Uses an alternate device*
40.	Shakoor, S., et al. (2019). "Common alternative diagnoses among a pediatric hospital-based cohort evaluated for tuberculosis in Karachi, Pakistan: The need	*Uses an alternate device*

	for facilitated referral in tuberculosis clinics." International Journal of Mycobacteriology 8(1): 42-47.	
41.	Stringer, M. (2021). "Active pulmonary tuberculosis presenting as post-menopausal bleeding." Australian and New Zealand Journal of Obstetrics and Gynaecology 61(SUPPL 1): 115.	*Uses an alternate device*
42.	Williams, B., et al. (2019). "Infection screening in unaccompanied asylum-seeking children." Archives of Disease in Childhood 104: A111-A112.	*Uses an alternate device*
43.	Alonzi, T., et al. (2023). "Research tests for the diagnosis of tuberculosis infection." Expert Review of Molecular Diagnostics 23(9): 783-795.	*Duplicate article*
44.	Altawallbeh, G., et al. (2021). "Performance of an Advanced Interferon-Gamma Release Assay for Mycobacterium tuberculosis Detection." The journal of applied laboratory medicine 6(5): 1287-1292.	*Duplicate article*
45.	Bisognin, F., et al. (2020). "QuantiFERON-TB gold plus with chemiluminescence immunoassay: Do we need a higher cutoff?" Journal of Clinical Microbiology 58(10).	*Duplicate article*
46.	Buron, V. and N. Banaei (2023). "Inflated Gamma Interferon Response with QuantiFERON-TB Gold Plus Using the Automated Liaison XL Analyzer: a Testing Algorithm To Mitigate False-Positive Results in Low-Incidence Settings." Journal of Clinical Microbiology 61(6): e0029523.	*Duplicate article*
47.	Buron, V. and N. Banaei (2023)."Erratum: Inflated Gamma Interferon Response with QuantiFERON-TB Gold Plus Using the Automated LIAISON XL Analyzer: a Testing Algorithm To Mitigate False-Positive Results in Low-Incidence Settings (Journal of Clinical Microbiology (2023) 61:6 (e00295-23) DOI: 10.1128/jcm.00295-23)." Journal of Clinical Microbiology 61(11).	*Duplicate article*
48.	Busà, R., et al. (2022). "Specific Anti-SARS-CoV-2 Humoral and Cellular Immune Responses After Booster Dose of BNT162b2 Pfizer-BioNTech mRNA-Based Vaccine: Integrated Study of Adaptive Immune System Components." Frontiers in Immunology 13.	*Duplicate article*
49.	Cornaby, C., et al. (2022). "Repeatability of QuantiFERON-TB gold plus testing utilizing microparticle chemiluminescence." Journal of Immunological Methods 509.	*Duplicate article*
50.	Cortez, R., et al. (2024). "Evaluation of the Use of One-tube Blood Collection and Automated Specimen Processing System for LIAISON® QuantiFERON®-TB Gold Plus Testing at a Large Regional Reference Laboratory." Clinical Chemistry 70: i96.	*Duplicate article*
51.	de Maertelaere, E., et al. (2020). "Evaluation of QuantiFERON-TB gold plus on Liaison XL in a low-tuberculosis-incidence setting." Journal of Clinical Microbiology 58(4).	*Duplicate article*
52.	Desmecht, S., et al. (2022). "Kinetics and Persistence of the Cellular and Humoral Immune Responses to BNT162b2 mRNA Vaccine in SARS-CoV-2-Naive and -Experienced Subjects: Impact of Booster Dose and Breakthrough Infections." Frontiers in Immunology 13.	*Duplicate article*
53.	Escalante, P., et al. (2023). "New diagnostics for the spectrum of asymptomatic TB: from infection to subclinical disease." International Journal of Tuberculosis and Lung Disease 27(7): 499-505.	*Duplicate article*
54.	Fernández-Huerta, M., et al. (2021). "Evaluation of the Fully Automated Chemiluminescence Analyzer LIAISON XL for the Performance of the QuantiFERON-TB Gold Plus Assay in an Area with a Low Incidence of Tuberculosis." Journal of Clinical Microbiology 59(8).	*Duplicate article*
55.	Fernández-Moreno, R., et al. (2024). "QuantiFERON–CMV assay by chemiluminescence immunoassay: Is it more suitable for real-live monitoring of transplant patients?" Journal of Clinical Virology 171.	*Duplicate article*
56.	Geller, J. (2020). "US Food and Drug Administration Announces Availability of Important Final Guidance." Journal of Clinical Engineering 45(2): 103-109.	*Duplicate article*
57.	Gong, W. and X. Wu (2021). "Differential Diagnosis of Latent Tuberculosis Infection and Active Tuberculosis: A Key to a Successful Tuberculosis Control Strategy." Frontiers in Microbiology 12.	*Duplicate article*
58.	Grassi, B., et al. (2019). "LIAISON®QUANTIFERON®-TB Gold Plus: A new fully automated interferon-gamma detection CLIA assay." Clinica Chimica Acta 493: S551-S552.	*Duplicate article*
59.	Hamada, Y., et al. (2021). "Tests for tuberculosis infection: Landscape analysis." European Respiratory Journal 58(5).	*Duplicate article*

60.	Heireman, L., et al. (2022). "Comparison of the QuantiFERON-TB® Gold Plus on LIAISON® XL and T-SPOT.TB for the diagnosis of latent Mycobacterium tuberculosis infection in a low tuberculosis incidence population." Diagnostic Microbiology and Infectious Disease 102(3).	*Duplicate article*
61.	Kadkhoda, K., et al. (2020). "Operational usability evaluation of the LIAISON® QuantiFERON®TB gold plus solution in a high volume laboratory setting." Clinical Laboratory 66(11): 2389-2390.	*Duplicate article*
62.	Kadkhoda, K., et al. (2022). Comparison of LIAISON QuantiFERON-TB Gold Plus with QuantiFERON-TB Gold Plus.	*Duplicate article*
63.	Kadkhoda, K., et al. (2023). "Comparison of LIAISON QuantiFERON-TB Gold Plus with QuantiFERON-TB Gold Plus." Microbiology Spectrum 11(1).	*Duplicate article*
64.	Khoury, R., et al. (2023). "Performance Evaluation of LIAISON® QuantiFERON®-TB Gold Plus Assay and Its Use in Long-Term Care Facilities." Clinical Chemistry 69: i99.	*Duplicate article*
65.	Kobashi, Y. (2023). "Current status and future landscape of diagnosing tuberculosis infection." Respiratory Investigation 61(5): 563-578.	*Duplicate article*
66.	Labdon, C. (2019). "LIAISON QuantiFERON-TB gold plus: Automation in LTBI testing." Clinica Chimica Acta 493: S775.	*Duplicate article*
67.	Mafi, S., et al. (2023). "Evaluation of the fully automated LIAISON®XL chemiluminescence analyzer for QuantiFERON®-CMV testing in transplant recipients." Journal of Clinical Virology 166.	*Duplicate article*
68.	Masiá, M., et al. (2023). "Integrating SARS-CoV-2-specific interferon-γ release assay testing in the evaluation of patients hospitalized with COVID-19." Microbiology Spectrum 11(6).	*Duplicate article*
69.	Mayorga Ayala, L. F., et al. (2022). "T cell response to SARS-CoV-2 mRNA vaccines by an interferon-gamma release immunoassay in patients with Inflammatory Bowel disease receiving anti-TNF and thiopurine treatment." Journal of Crohn's and Colitis 16: i525.	*Duplicate article*
70.	Mehreen, A., et al. (2023). "Investigation of High Rate of Low Positive QuantiFERON-TB Gold Plus Results." Clinical Chemistry 69: i61-i62.	*Duplicate article*
71.	Prestileo, T., et al. (2021). "Tuberculosis among migrant populations in sicily: A field report." Journal of Tropical Medicine 2021.	*Duplicate article*
72.	Ruiz-Tagle, C., et al. (2024). "Evaluation of concordance of new QuantiFERON-TB Gold Plus platforms for Mycobacterium tuberculosis infection diagnosis in a prospective cohort of household contacts." Microbiology Spectrum 12(8).	*Duplicate article*
73.	Stojkovic, V., et al. (2019). "Evaluation of DiaSorin QuantiFERON-TB Gold Plus test using LIAISON XL." Clinica Chimica Acta 493: S561.	*Duplicate article*
74.	Altawallbeh, G., et al. (2021). "Performance of an Advanced Interferon-Gamma Release Assay for Mycobacterium tuberculosis Detection." The journal of applied laboratory medicine 6(5): 1287-1292.	*Duplicate article*
75.	Bisognin, F., et al. (2020). "QuantiFERON-TB gold plus with chemiluminescence immunoassay: Do we need a higher cutoff?" Journal of Clinical Microbiology 58(10).	*Duplicate article*
76.	Buron, V. and N. Banaei (2023). "Inflated Gamma Interferon Response with QuantiFERON-TB Gold Plus Using the Automated LIAISONXL Analyzer: a Testing Algorithm To Mitigate False-Positive Results in Low-Incidence Settings." Journal of Clinical Microbiology 61(6): e0029523.	*Duplicate article*
77.	Buron, V. and N. Banaei (2023)."Erratum: Inflated Gamma Interferon Response with QuantiFERON-TB Gold Plus Using the Automated LIAISON XL Analyzer: a Testing Algorithm To Mitigate False-Positive Results in Low-Incidence Settings (Journal of Clinical Microbiology (2023) 61:6 (e00295-23) DOI: 10.1128/jcm.00295-23)." Journal of Clinical Microbiology 61(11).	*Duplicate article*
78.	Cave, L., et al. (2020). "Levelling Education Outcomes for Students With Medical and Mental Health Needs." Contin Educ 1(1): 98-117.	*Does not report on the intended patient population*
79.	Cornaby, C., et al. (2022). "Repeatability of QuantiFERON-TB gold plus testing utilizing microparticle chemiluminescence." Journal of Immunological Methods 509.	*Duplicate article*
80.	de Maertelaere, E., et al. (2020). "Evaluation of QuantiFERON-TB gold plus on Liaison XL in a low-tuberculosis-incidence setting." Journal of Clinical Microbiology 58(4).	*Duplicate article*

81.	Fernández-Huerta, M., et al. (2021). "Evaluation of the Fully Automated Chemiluminescence Analyzer LIAISON XL for the Performance of the QuantiFERON-TB Gold Plus Assay in an Area with a Low Incidence of Tuberculosis." Journal of Clinical Microbiology 59(8).	*Duplicate article*
82.	Fernández-Moreno, R., et al. (2024). "QuantiFERON–CMV assay by chemiluminescence immunoassay: Is it more suitable for real-live monitoring of transplant patients?" Journal of Clinical Virology 171.	*Duplicate article*
83.	Gallini, N., et al. (2023). "Implementation and impact of the global access principles at the University of British Columbia: current successes and future challenges." Frontiers in Pharmacology 14.	*Duplicate article*
84.	Gong, W. and X. Wu (2021). "Differential Diagnosis of Latent Tuberculosis Infection and Active Tuberculosis: A Key to a Successful Tuberculosis Control Strategy." Frontiers in Microbiology 12.	*Duplicate article*
85.	Haley, C. A., et al. (2023). "Implementation of Bedaquiline, Pretomanid, and Linezolid in the United States: Experience Using a Novel All-Oral Treatment Regimen for Treatment of Rifampin-Resistant or Rifampin-Intolerant Tuberculosis Disease." Clin Infect Dis 77(7): 1053-1062.	*Uses an alternate device*
86.	Heireman, L., et al. (2022). "Comparison of the QuantiFERON-TB® Gold Plus on LIAISON® XL and T-SPOT.TB for the diagnosis of latent Mycobacterium tuberculosis infection in a low tuberculosis incidence population." Diagnostic Microbiology and Infectious Disease 102(3).	*Duplicate article*
87.	Hervig, M. E., et al. (2024). "5-HT 2A and 5-HT 2C receptor antagonism differentially modulate reinforcement learning and cognitive flexibility: behavioural and computational evidence." Psychopharmacology (Berl) 241(8): 1631-1644.	*Does not report on the intended patient population*
88.	Jafri, S., et al. (2019). "LIAISON OF SUGAR CONTROL WITH TIME TO SPUTUM SMEAR CONVERSION IN MULTIDRUG-RESISTANT TB." Chest 156(4): A362.	*Duplicate article*
89.	Jain, M., et al. (2024). "Using a quality improvement tool, Plan-Do-Study-Act cycle, to boost TB notification in India post-Covid-19 pandemic." Indian J Tuberc 71(3): 360-365.	*Uses an alternate device*
90.	Kadkhoda, K., et al. (2020). "Operational usability evaluation of the LIAISON® QuantiFERON®TB gold plus solution in a high volume laboratory setting." Clinical Laboratory 66(11): 2389-2390.	*Duplicate article*
91.	Kadkhoda, K., et al. (2023). "Comparison of LIAISON QuantiFERON-TB Gold Plus with QuantiFERON-TB Gold Plus." Microbiology Spectrum 11(1).	*Duplicate article*
92.	Kobayashi, Y., et al. (2022). "Multidrug-Resistant Tuberculosis Outbreak among Immigrants in Tokyo, Japan, 2019–2021." Japanese Journal of Infectious Diseases 75(5): 527-529.	*Duplicate article*
93.	Lampaert, E., et al. (2024). "Evaluation of centralised and decentralised models of care during the 2020 Ebola Virus Disease outbreak in Equateur Province, Democratic Republic of the Congo: A brief report." F1000Research 13.	*Duplicate article*
94.	Müller, M. P., et al. (2024). "Reporting standard for describing first responder systems, smartphone alerting systems, and AED networks." Resuscitation 195: 110087.	*Does not report on the intended patient population*
95.	Nallamothu, B. K., et al. (2023). "Ten Steps Toward Improving In-Hospital Cardiac Arrest Quality of Care and Outcomes." Circ Cardiovasc Qual Outcomes 16(11): e010491.	*Does not report on the intended patient population*
96.	Rakshit, S., et al. (2022). "Evidence for the heterologous benefits of prior BCG vaccination on COVISHIELD™ vaccine-induced immune responses in SARS-CoV-2 seronegative young Indian adults." Frontiers in Immunology 13.	*Duplicate article*
97.	Rosenfeld, R. M., et al. (2022). "Dietary Interventions to Treat Type 2 Diabetes in Adults with a Goal of Remission: An Expert Consensus Statement from the American College of Lifestyle Medicine." Am J Lifestyle Med 16(3): 342-362.	*Does not report on the intended patient population*
98.	Ruiz-Tagle, C., et al. (2024). "Evaluation of concordance of new QuantiFERON-TB Gold Plus platforms for Mycobacterium tuberculosis infection diagnosis in a prospective cohort of household contacts." Microbiology Spectrum 12(8).	*Duplicate article*
99.	Shakoor, S., et al. (2019). "Common alternative diagnoses among a pediatric hospital-based cohort evaluated for tuberculosis in Karachi, Pakistan: The need for facilitated referral in tuberculosis clinics." International Journal of Mycobacteriology 8(1): 42-47.	*Duplicate article*

100.	Sutar, R., et al. (2024). "Anxiety, stress, and quality of life in patients with tuberculosis: A systematic review and meta-analysis." Ind Psychiatry J 33(1): 13-29.	*Uses an alternate device*
101.	Wang, C., et al. (2023). "Coronary CT Angiography-based Morphologic Index for Predicting Hemodynamically Significant Coronary Stenosis." Radiol Cardiothorac Imaging 5(6): e230064.	*Does not report on the intended patient population*
102.	Wortham, J. M., et al. (2024). "Second Nationwide Tuberculosis Outbreak Caused by Bone Allografts Containing Live Cells - United States, 2023." MMWR Morb Mortal Wkly Rep 72(5253): 1385-1389.	*Uses an alternate device*
103.	Abella, L., et al. (2023). "Early changes in S100B maternal blood levels can predict fetal intrauterine growth restriction." Clinical Chemistry and Laboratory Medicine (CCLM) 61(12): 2205-2211.	*Does not report on the intended patient population*
104.	Alfi, O., et al. (2024). "Decidual-tissue-resident memory T cells protect against nonprimary human cytomegalovirus infection at the maternal-fetal interface." Cell Reports 43(2).	*Does not report on the intended patient population*
105.	Alonzi, T., et al. (2023). "Research tests for the diagnosis of tuberculosis infection." Expert Review of Molecular Diagnostics 23(9): 783-795.	*Duplicate article*
106.	Altawallbeh, G., et al. (2021). "Performance of an Advanced Interferon-Gamma Release Assay for Mycobacterium tuberculosis Detection." The journal of applied laboratory medicine 6(5): 1287-1292.	*Duplicate article*
107.	Anaïs, D. (2019). "Sous la direction de M. MAHAZA Chetaou."	*Not from a peer-reviewed source*
108.	Asnawi, A. W. B. A., et al. (2022). "5th USIM International Health Conference 2022, Lessons from pandemic: Reshaping Medicine and Healthcare." Malaysian Journal of Public Health Medicine 22(Suppl. 2).	*Uses an alternate device*
109.	Barreiro, P., et al. (2022). "A pilot study for the evaluation of an interferon gamma release assay (IGRA) to measure T-cell immune responses after SARS-CoV-2 infection or vaccination in a unique cloistered cohort." Journal of Clinical Microbiology 60(3): e02199-02121.	*Does not report on the intended patient population*
110.	Bisognin, F., et al. (2020). "QuantiFERON-TB Gold Plus with chemiluminescence immunoassay: do we need a higher cutoff?" Journal of Clinical Microbiology 58(10): 10.1128/jcm. 00780-00720.	*Duplicate article*
111.	Buron, V. and N. Banaei (2023). "Inflated gamma interferon response with QuantiFERON-TB Gold Plus using the automated LIAISON XL analyzer: a testing algorithm to mitigate false-positive results in low-incidence settings." Journal of Clinical Microbiology 61(6): e00295-00223.	*Duplicate article*
112.	Buron, V. and N. Banaei (2023). "Erratum for Buron and Banaei,“Inflated Gamma Interferon Response with QuantiFERON-TB Gold Plus Using the Automated LIAISON XL Analyzer: a Testing Algorithm To Mitigate False-Positive Results in Low-Incidence Settings”." Journal of Clinical Microbiology 61(11): e01067-01023.	*Duplicate article*
113.	Busà, R., et al. (2022). "Specific anti-SARS-CoV-2 humoral and cellular immune responses after booster dose of BNT162b2 pfizer-BioNTech mRNA-based vaccine: Integrated study of adaptive immune system components." Frontiers in Immunology 13: 856657.	*Duplicate article*
114.	CARLETTI, A. (2019). "Appropriatezza prescrittiva del test QuantiFERON-TB Gold Plus nell'Azienda Ospedaliera Ospedali Riuniti Marche Nord."	*Not from a peer-reviewed source*
115.	Castellotti, P., et al. (2020). "Intestinal tuberculosis versus Inflammatory Bowel Diseases: a never-ending challenge." New Microbiol 43(3): 139-143.	*Case report*
116.	Cheng, P., et al. (2022). "In silico analysis of peptide-based biomarkers for the diagnosis and prevention of latent tuberculosis infection." Frontiers in microbiology 13: 947852.	*Uses an alternate device*
117.	Chongxing, Z., et al. (2023). "Evaluation of the diagnostic efficacy of EC-Test for latent tuberculosis infection in ambulatory people with HIV." Aids 37(12): 1791-1797.	*Uses an alternate device*
118.	Coignard, C., et al. (2019). "Evaluation of the BioPlex 2200 Lyme IgG/IgM immunoassay system." Clin. Chim. Acta 493: 560-566.	*Does not report on the intended patient population*
119.	Colombini, A., et al. (2023). "The total testing process harmonization: the case study of SARS-CoV-2 serological tests." Clinical Chemistry and Laboratory Medicine (CCLM) 61(12): 2084-2093.	*Does not report on the intended patient population*
120.	Comella-del-Barrio, P., et al. (2019). "A model based on the combination of IFN-γ, IP-10, ferritin and 25-hydroxyvitamin D for discriminating latent from active tuberculosis in children." Frontiers in microbiology 10: 1855.	*Uses an alternate device*

121.	Cornaby, C., et al. (2022). "Repeatability of QuantiFERON-TB Gold Plus testing utilizing microparticle chemiluminescence." Journal of Immunological Methods 509: 113340.	*Duplicate article*
122.	Cortez, R., et al. (2024). "A-252 Evaluation of the Use of One-tube Blood Collection and Automated Specimen Processing System for LIAISON® QuantiFERON®-TB Gold Plus Testing at a Large Regional Reference Laboratory." Clinical Chemistry 70(Supplement_1): hvae106. 249.	*Duplicate article*
123.	Crespo, M., et al. (2022). "Negative immune responses to two-dose mRNA COVID-19 vaccines in renal allograft recipients assessed with simple antibody and interferon gamma release assay cellular monitoring." American Journal of Transplantation 22(3): 786-800.	*Does not report on the intended patient population*
124.	Culebras, E., et al. (2024). "Cell immunity to SARS-CoV-2 after natural infection and/or different vaccination regimens." Frontiers in Cellular and Infection Microbiology 14: 1370859.	*Does not report on the intended patient population*
125.	de Maertelaere, E., et al. (2020). "Evaluation of QuantiFERON-TB Gold Plus on LIAISON XL in a low-tuberculosis-incidence setting." Journal of Clinical Microbiology 58(4): 10.1128/jcm. 00159-00120.	*Duplicate article*
126.	Desmecht, S., et al. (2022). "Pere e H, Tokunaga Y, Fombellida-Lopez C, Polese B, Legrand C, Wery M, Mni M, Fouillien N, Toussaint F, Gillet L, Bureau F, Lutteri L, Hayette MP, Moutschen M, Meuris C, Vermeersch P, Desmecht D, Rahmouni S and Darcis G (2022) Kinetics and Persistence of the Cellular and Humoral Immune Responses to BNT162b2 mRNA Vaccine in SARS-CoV-2-Naive and-Experienced Subjects: Impact of Booster Dose and Breakthrough Infections." Front. Immunol. 13: 863554. doi: 10.3389/fimmu.	*Duplicate article*
127.	Desmecht, S., et al. (2022). "Kinetics and persistence of the cellular and humoral immune responses to BNT162b2 mRNA vaccine in SARS-CoV-2-naive and-experienced subjects: impact of booster dose and breakthrough infections." Frontiers in Immunology 13: 863554.	*Duplicate article*
128.	Dewi, J. (2024). "Perbandingan Uji TB IGRA Menggunakan Metode T-SPOT. TB dan CLIA." MEDICINUS 37(3): 22-26.	*Uses an alternate device*
129.	Diel, R., et al. (2023). "Empfehlungen für die Umgebungsuntersuchungen bei Tuberkulose–Update 2023." Pneumologie 77(09): 607-631.	*Not available in English after reasonable attempts have been made to translate the article to determine applicability.*
130.	Eisinger, R. W., et al. (2020). "2019: a banner year for tuberculosis research." The Journal of Infectious Diseases 222(11): 1768-1771.	*Uses an alternate device*
131.	El Moussaoui, M., et al. (2023). "Cluster analysis identifies distinct patterns of T-cell and humoral immune responses evolution following a third dose of SARS-CoV-2 vaccine in people living with HIV." Viruses 15(7): 1435.	*Does not report on the intended patient population*
132.	Eliška, S. (2024). Porovnání metody Quantiferon TB-gold a stanovení aktivačních markerů na T-lymfocytech pomocí průtokové cytometrie, České vysoké učení technické v Praze. Vypočetní a informační centrum.	*Not from a peer-reviewed source*
133.	Etienne, P. "Histoire et anthropologie de la vie sociale d'une catégorie diagnostique: l'Infection Tuberculeuse Latente."	*Not from a peer-reviewed source*
134.	Fangous, M., et al. (2020). "QuantiFERON et Liaison XL: retour d'expérience sur les tests indéterminés." Médecine et Maladies Infectieuses 50(6): S152.	*Not available in English after reasonable attempts have been made to translate the article to determine applicability.*
135.	Fernández-González, M., et al. (2023). "T-cell immunity against severe acute respiratory syndrome coronavirus 2 measured by an interferon-γ release assay is strongly associated with patient outcomes in vaccinated persons hospitalized with Delta or Omicron variants." The Journal of Infectious Diseases 228(9): 1240-1252.	*Does not report on the intended patient population*
136.	Fernández-Huerta, M., et al. (2021). "Evaluation of the fully automated chemiluminescence analyzer LIAISON XL for the performance of the QuantiFERON-TB Gold Plus assay in an area with a low incidence of tuberculosis." Journal of Clinical Microbiology 59(8): 10.1128/jcm. 00603-00621.	*Duplicate article*
137.	Fernández-Huerta, M., et al. (2020). "Clinical evaluation of LIAISON XL for the performance of the QuantiFERON-TB Gold Plus assay in a low-incidence tuberculosis area" Rev Enf Emerg;19(3):206-211.	Duplicated record

138.	Fernández-Moreno, R., et al. (2024). "QuantiFERON–CMV assay by chemiluminescence immunoassay: Is it more suitable for real-live monitoring of transplant patients?" Journal of Clinical Virology 171: 105651.	*Duplicate article*
139.	García Gasalla, M., et al. (2023). "Reversions of QuantiFERON-TB Gold Plus in tuberculosis contact investigation: A prospective multicentre cohort study." PLoS One, 2023, vol. 18, num. 8.	*Primarily contains duplicate data from another included study*
140.	Gatti, A., et al. (2020). "Quantification of neutrophil and monocyte CD64 expression: a predictive biomarker for active tuberculosis." The International Journal of Tuberculosis and Lung Disease 24(2): 196-201.	*Does not report on the intended patient population*
141.	Geller, J. (2020). "US Food and Drug Administration Announces Availability of Important Final Guidance." Journal of Clinical Engineering 45(2): 103-109.	*Duplicate article*
142.	Goletti, D., et al. (2022). "The role of IGRA in the diagnosis of tuberculosis infection, differentiating from active tuberculosis, and decision making for initiating treatment or preventive therapy of tuberculosis infection." International Journal of Infectious Diseases 124: S12-S19.	Narrative review
143.	Gong, W., et al. (2022). "Prediction of Th1 and cytotoxic T lymphocyte epitopes of Mycobacterium tuberculosis and evaluation of their potential in the diagnosis of tuberculosis in a mouse model and in humans." Microbiology Spectrum 10(4): e01438-01422.	*Does not report on the intended patient population*
144.	Gong, W. and X. Wu (2021). "Differential diagnosis of latent tuberculosis infection and active tuberculosis: a key to a successful tuberculosis control strategy." Frontiers in microbiology 12: 745592.	*Duplicate article*
145.	Granozzi, B., et al. (2022). "IGRA test for TB in COVID-19: role of corticosteroids." The International Journal of Tuberculosis and Lung Disease 26(11): 1088-1091.	*Uses an alternate device*
146.	Grassi, B., et al. (2019). "LIAISON® QUANTIFERON®-TB Gold Plus: A new fully automated interferon-gamma detection CLIA assay." Clinica Chimica Acta 493: S551-S552.	*Duplicate article*
147.	Greco, M., et al. (2021). "Human leukocyte antigen-DR isotype expression in monocytes and T cells interferon-gamma release assay in septic patients and correlation with clinical outcome." Journal of clinical medicine research 13(5): 293.	*Does not report on the intended patient population*
148.	Haggenburg, S., et al. (2022). SARS-CoV-2 vaccine-induced humoral and cellular immunity in patients with hematologic malignancies. Seminars in Hematology, Elsevier.	*Does not report on the intended patient population*
149.	Hattingh, C. D., et al. "The effects of a combined exercise intervention on gut microbiomes and systemic inflammatory biomarkers in NAFLD patients."	*Does not report on the intended patient population*
150.	Havelkaa, A., et al. (2019). "From niche to full automation–State of the art management for workflow optimization." Abstracts/Clinica Chimica Acta 493: S763-S776.	*Uses an alternate device*
151.	Hazra, R. S., et al. (2022). "Bioinspired materials for wearable devices and point-of-care testing of cancer." ACS biomaterials science & engineering 9(5): 2103-2128.	*Does not report on the intended patient population*
152.	Heidary, M., et al. (2022). "Tuberculosis challenges: Resistance, co-infection, diagnosis, and treatment." European Journal of Microbiology and Immunology 12(1): 1-17.	Narrative review
153.	Heireman, L., et al. (2022). "Comparison of the QuantiFERON-TB® Gold Plus on LIAISON® XL and T-SPOT. TB for the diagnosis of latent Mycobacterium tuberculosis infection in a low tuberculosis incidence population." Diagnostic microbiology and infectious disease 102(3): 115613.	*Duplicate article*
154.	Heyd, A. T. (2020). "Management of Latent Tuberculosis Infection Among an Inner-city Population with Psychosocial Barriers to Treatment Adherence."	*Not from a peer-reviewed source*
155.	Jimenez Balarezo, M., et al. (2022). "Cellular and humoral immunogenicity of the mRNA-1273 SARS-CoV-2 vaccine in patients with hematologic malignancies."	*Does not report on the intended patient population*
156.	Jiménez, M., et al. (2023). "Kinetics of cellular and humoral immunogenicity and effectiveness of SARS-CoV-2 booster vaccination in hematologic neoplasms." American Journal of Hematology 98(8): 1204-1213.	*Does not report on the intended patient population*
157.	Jomehpour, N., et al. (2023). "Diagnosis of Latent Tuberculosis Infection: Promising Antigens." Iranian Journal of Medical Microbiology 17(5): 506-519.	*Not available for download or purchase after reasonable attempts have been made*

158.	Kadkhoda, K., et al. (2020). "Operational Usability Evaluation of the LIAISON® QuantiFERON®-TB Gold Plus Solution in a High Volume Laboratory Setting." Clinical Laboratory(11).	*Duplicate article*
159.	Khoury, R., et al. (2023). "A-285 Performance Evaluation of LIAISON® QuantiFERON®-TB Gold Plus Assay and Its Use in Long-Term Care Facilities." Clinical Chemistry 69(Supplement_1): hvad097. 250.	*Duplicate article*
160.	Kobashi, Y. (2023). "Current status and future landscape of diagnosing tuberculosis infection." Respiratory Investigation 61(5): 563-578.	*Duplicate article*
161.	Kontsevaya, I., et al. (2023). "Update on the diagnosis of tuberculosis." Clinical Microbiology and Infection.	Narrative review
162.	Korventausta, N. and J. Salonen (2022). "QuantiFERON®-TB GOLD PLUS–IGRA-testin verifiointi ja käyttöönotto."	*Not available in English after reasonable attempts have been made to translate the article to determine applicability.*
163.	Krüttgen, A., et al. (2021). "Evaluation of the QuantiFERON SARS-CoV-2 interferon-ɣ release assay in mRNA-1273 vaccinated health care workers." Journal of Virological Methods 298: 114295.	*Does not report on the intended patient population*
164.	Labdon, C. (2019). "LIAISON QuantiFERON-TB gold plus: Automation in LTBI testing." Clinica Chimica Acta 493: S775.	*Duplicate article*
165.	Lairmore, S., et al. (2021). "Infectious disease screening in a dedicated primary care clinic for children in foster care." Child Abuse & Neglect 117: 105074.	*Does not report on the intended patient population*
166.	Longet, S., et al. (2023). "Rosalia Busà1, Maria Concetta Sorrentino2, Giovanna Russelli1, Giandomenico Amico1, 3, Vitale Miceli1, Monica Miele1, 3, Mariangela Di Bella1, 3, Francesca Timoneri1, 3, Alessia Gallo1, Giovanni Zito1, Daniele Di Carlo2, Pier Giulio Conaldi1 and Matteo Bulati1." Innate immune responses to SARS-CoV-2 in infected and vaccinated individuals 16648714: 39.	*Does not report on the intended patient population*
167.	LUTTERI, L. (2019). LIAISON QuantiFERON TB Gold Plus: customer experience. Congrès Diasorin.	*Not from a peer-reviewed source*
168.	Mafi, S., et al. (2023). "Evaluation of the fully automated LIAISON® XL chemiluminescence analyzer for QuantiFERON®-CMV testing in transplant recipients." Journal of Clinical Virology 166: 105550.	*Duplicate article*
169.	Magicová, M. (2024). "Aspekty imunitní odpovědi u pacientů po transplantaci ledviny po infekci virem SARS-CoV-2."	*Not from a peer-reviewed source*
170.	Magicova, M., et al. (2022). "Determinants of immune response to anti–SARS-CoV-2 mRNA vaccines in kidney transplant recipients: a prospective cohort study." Transplantation 106(4): 842-852.	*Does not report on the intended patient population*
171.	Mahmoudi, S. and S. M. S. Hosseini Sharif (2024). "Diagnostic accuracy of QuantiFERON-TB Gold Plus with Chemiluminescence Immunoassay: a systematic review and meta-analysis." Expert Review of Clinical Immunology: 1-10.	*Uses an alternate device*
172.	Mahmoudi, S., et al. (2023). Immunodiagnostics of tuberculosis: recent discoveries. Tuberculosis: Integrated Studies for a Complex Disease, Springer: 139-150.	*Not from a peer-reviewed source*
173.	Maipan-Uku, J. Y. and N. Cavus (2024). "Forecasting tuberculosis incidence: a review of time series and machine learning models for prediction and eradication strategies." International Journal of Environmental Health Research: 1-16.	*Uses an alternate device*
174.	Masiá, M., et al. (2023). "Integrating SARS-CoV-2-specific interferon-γ release assay testing in the evaluation of patients hospitalized with COVID-19." Microbiology Spectrum 11(6): e02419-02423.	*Duplicate article*
175.	Mayorga Ayala, L., et al. (2022). "P588 T cell response to SARS-CoV-2 mRNA vaccines by an interferon-gamma release immunoassay in patients with inflammatory bowel disease receiving anti-TNF and thiopurine treatment." Journal of Crohn's and Colitis 16(Supplement_1): i525-i525.	*Duplicate article*
176.	Mehreen, A., et al. (2023). "A-170 Investigation of High Rate of Low Positive Quantiferon-TB Gold Plus Results." Clinical Chemistry 69(Supplement_1): hvad097. 154.	*Duplicate article*
177.	Mendelsohn, S. C., et al. (2022). "Clinical predictors of pulmonary tuberculosis among South African adults with HIV." EClinicalMedicine 45.	*Uses an alternate device*

178.	Mendelsohn, S. C., et al. "Prospective Validation of a Host Blood Transcriptomic Biomarker for Pulmonary Tuberculosis in People Living with HIV: A Diagnostic and Prognostic Accuracy Study."	*Uses an alternate device*
179.	Migliori, G. B., et al. (2021). "The definition of tuberculosis infection based on the spectrum of tuberculosis disease." Breathe 17(3).	Narrative review
180.	Mouton, W. (2022). Etude des altérations fonctionnelles du système immunitaire des sujets immunodéprimés, Université de Lyon.	*Not from a peer-reviewed source*
181.	Nadeau, K. (2022). "Selecting the right analyzer technology to meet your laboratory's testing needs." Medical Laboratory Observer 54(6): 32-35.	*Does not report on the intended patient population*
182.	Nolt, D., et al. (2021). "Tuberculosis infection in children and adolescents: testing and treatment." Pediatrics 148(6).	*Uses an alternate device*
183.	Oliver, S. J. (2022). "From Isolation to Inclusion: Embracing Local Perspectives in Examining the Treatment Model of Care for Aboriginal Persons Affected by Tuberculosis or Leprosy in the Kimberley Region, North Western Australia."	*Not from a peer-reviewed source*
184.	Ortiz-Brizuela, E., et al. (2023). "Assessing the diagnostic performance of new commercial interferon-γ release assays for Mycobacterium tuberculosis infection: a systematic review and Meta-analysis." Clinical Infectious Diseases 76(11): 1989-1999.	Systematic review with studies already included
185.	Ozaki, B., et al. (2024). "A-250 Evaluation of Liver Fibrosis in Schistosomiasis Mansoni Using the ELF (Enhanced Liver Fibrosis) Score." Clinical Chemistry 70(Supplement_1): hvae106. 247.	*Uses an alternate device*
186.	Pagaduan, J. V. and G. Altawallbeh (2023). Advances in TB testing. Advances in Clinical Chemistry, Elsevier. 115: 33-62.	*Uses an alternate device*
187.	Pagnoncelli, M., et al. (2024). "Performance of the T-SPOT. TB test in patients with indeterminate QuantiFERON-TB Gold Plus results: proposal for an algorithm for the diagnosis of Latent Tuberculosis Infection." Le Infezioni in Medicina 32(4): 525.	*Uses an alternate device*
188.	Palacios-Gutiérrez, J.-J., et al. (2022). "Clinical and epidemiological correlates of low IFN-gamma responses in mitogen tube of QuantiFERON assay in tuberculosis infection screening during the COVID-19 pandemic: a population-based marker of COVID-19 mortality?" Archivos de Bronconeumologia 58(9): 649-659.	*Uses an alternate device*
189.	Parvez, F. M. (2022). "Prevention and control of tuberculosis in correctional facilities." Public health behind bars: from prisons to communities: 157-193.	*Uses an alternate device*
190.	Patel, P. "Can medical laboratories give humanity the edge over tuberculosis?".	*Uses an alternate device*
191.	Peregrina Rivas, J. A. "Impacto de la utilización de fármacos inmunosupresores en la covid-19 sobre la reactivación de infección tuberculosa latente y virus de la Hepatitis B."	*Not from a peer-reviewed source*
192.	Pérez-Recio, S., et al. (2023). "Reversions of QuantiFERON-TB Gold Plus in tuberculosis contact investigation: A prospective multicentre cohort study." Plos one 18(8): e0285917.	*Uses an alternate device*
193.	Prestileo, T., et al. (2021). "Tuberculosis among Migrant Populations in Sicily: A Field Report." Journal of tropical medicine 2021(1): 7856347.	*Duplicate article*
194.	Puyana Ortiz, J. D., et al. (2023). "Adherence and toxicity during the treatment of latent tuberculous infection in a referral center in Spain." Tropical Medicine and Infectious Disease 8(7): 373.	No comparison done
195.	Ramadan-Boscolo, D., et al. (2024). "A-253 Antimicrobial Susceptibility Pattern in Escherichia coli Isolated From Community-Acquired Urinary Tract Infections in the era Post-Covid-19." Clinical Chemistry 70(Supplement_1): hvae106. 250.	*Does not report on the intended patient population*
196.	Rothoeft, T., et al. (2024). "Natural and hybrid immunity after SARS-CoV-2 infection in children and adolescents." Infection: 1-10.	*Does not report on the intended patient population*
197.	Rudeeaneksin, J., et al. (2023). "QuantiFERON-TB Gold Plus and QuantiFERON-TB Gold In-tube assays for detecting latent tuberculosis infection in Thai healthcare workers." Revista do Instituto de Medicina Tropical de São Paulo 65: e13.	*Uses an alternate device*
198.	Ruiz-Tagle, C., et al. (2024). "Evaluation of concordance of new QuantiFERON-TB Gold Plus platforms for Mycobacterium tuberculosis infection diagnosis in a prospective cohort of household contacts." Microbiology Spectrum 12(8): e00469-00424.	*Duplicate article*

199.	San Román, J., et al. (2022). "Humoral and cellular response after mRNA vaccination in nursing homes: Influence of age and of history of COVID-19." Vaccines 10(3): 383.	*Does not report on the intended patient population*
200.	Scriba, T. J., et al. (2021). "Biomarker-guided tuberculosis preventive therapy (CORTIS): a randomised controlled trial." The Lancet Infectious Diseases 21(3): 354-365.	*Uses an alternate device*
201.	Seely, S., et al. (2024). "A-251 Simplification of Molecular Diagnostics for Sexually Transmitted Infection Point-of-Care Testing." Clinical Chemistry 70(Supplement_1): hvae106. 248.	*Does not report on the intended patient population*
202.	Sgherza, N., et al. (2024). "Latent tuberculosis infection detected by QuantiFERON-TB assay in patients with multiple myeloma receiving novel drugs: focus on reactivation prophylaxis in a retrospective, single-center study." Annals of Hematology: 1-3.	No comparison done
203.	Sierra, C. J. and C. A. Downs (2022). "Screening and testing for latent tuberculosis infection among patients who are immunocompromised." The Nurse Practitioner 47(11): 32-39.	This is a narrative review
204.	Song, J., et al. (2024). "A Retrospective Study of Factors Contributing to the Performance of an Interferon-Gamma Release Assay Blood Test for Tuberculosis Infection." Clinical Chemistry 70(3): 551-561.	*Uses an alternate device*
205.	STOJKOVIC, V., et al. (2019). Evaluation of Diasorin QuantiFERON-TB Gold Plus test using LIAISON XL. Euromedlab.	*Duplicate article*
206.	STOJKOVIC, V., et al. (2019). Evaluation of QuantiFERON TB Gold Plus Test Using LIAISON XL Analyzer. Annual Meeting of the Royal Belgian Society of Laboratory Medicine.	*Duplicate article*
207.	Sumner, T., et al. (2021). "The effect of new Mycobacterium tuberculosis infection on the sensitivity of prognostic TB signatures." The International Journal of Tuberculosis and Lung Disease 25(12): 1001-1005.	*Uses an alternate device*
208.	Sunny, P. (2024). The Lessons Learned from An Active Tuberculosis Genotyping Cluster Investigation in Allegheny County, University of Pittsburgh.	*Uses an alternate device*
209.	Tamhaev, R. (2024). Conception, synthèse et caractérisation de dérivés diaryl éthers comme nouveaux inhibiteurs directs de la protéine InhA de Mycobacterium tuberculosis, Université de Toulouse.	*Not from a peer-reviewed source*
210.	Timmer, A., et al. (2022). "P587 Disease severity in the INFLAMMATORY BOWEL DISEASES: Do patients and physicians agree?" Journal of Crohn's and Colitis 16(Supplement_1): i524-i525.	*Does not report on the intended patient population*
211.	TRANFA, A. (2022). "LIAISON QuantiFERON-TB Gold Plus: un nuovo approccio diagnostico alla tubercolosi latente."	*Not from a peer-reviewed source*
212.	TRUTH, A. M. O. "AVAILABLE THROUGH QUEST DIAGNOSTICS®."	*Uses an alternate device*
213.	TRUTH, A. M. O. "THE SCIENCE BEHIND THE T-SPOT®. TB TEST."	*Uses an alternate device*
214.	TRUTH, A. M. O. "THE T-SPOT®. TB TEST DIFFERENCE."	*Uses an alternate device*
215.	Villa, S., et al. (2019). "Latent tuberculosis infection treatment completion while shifting prescription from isoniazid-only to rifampicin-containing regimens: a two-decade experience in Milan, Italy." Journal of Clinical Medicine 9(1): 101.	Narrative review
216.	Ward, J. D., et al. (2021). "Indeterminate QuantiFERON gold plus results reveal deficient interferon gamma responses in severely ill COVID-19 patients." Journal of Clinical Microbiology 59(10): 10.1128/jcm. 00811-00821.	Uses an alternate device
217.	Wikell, A., et al. (2021). "The impact of borderline QuantiFERON-TB Gold Plus results for latent tuberculosis screening under routine conditions in a low-endemicity setting." Journal of Clinical Microbiology 59(12): 10.1128/jcm. 01370-01321.	*Uses an alternate device*
218.	Woelfel, S., et al. (2023). "Systemic and T cell-associated responses to SARS-CoV-2 immunisation in gut inflammation (STAR SIGN study): effects of biologics on vaccination efficacy of the third dose of mRNA vaccines against SARS-CoV-2." Alimentary pharmacology & therapeutics 57(1): 103-116.	*Does not report on the intended patient population*
219.	WHO (2022). "Use of alternative interferon-gamma release assays for the diagnosis of TB infection: WHO policy statement: web annex: study report."	*Uses an alternate device*
220.	Zabalza, A., et al. (2022). "Humoral and cellular responses to SARS-CoV-2 in convalescent COVID-19 patients with multiple sclerosis." Neurology: Neuroimmunology & Neuroinflammation 9(2): e1143.	*Does not report on the intended patient population*

221.	Zhou, C., et al. (2023). "Evaluation of the diagnostic efficacy of EC-test for latent tuberculosis infection in ambulatory people living with HIV."	*Uses an alternate device*
222.	Zhuang, L., et al. (2023). "Next-generation TB vaccines: progress, challenges, and prospects." Vaccines 11(8): 1304.	*Uses an alternate device*
