# Supplementary material for: Agreement and systematic bias between QuantiFERON chemiluminescent immunoassay and QuantiFERON enzyme-linked immunosorbent assay in the detection of latent tuberculosis infection: A systematic review and meta-analysis
Source: IJID Reg. 2025 Dec 7;18:100824. doi: 10.1016/j.ijregi.2025.100824 (PMC12809075; doi:10.1016/j.ijregi.2025.100824)
Supplement: Supplementary file 4 [file mmc4.pdf]

S2. Summary of Risk of Bias Assessments Across Included Studies (QUADAS-2 Tool)

Author	Year	DOI	Country	Patient Selection - Risk of Bias	Patient Selection - Applicability	Index Test - Risk of Bias	Index Test - Applicability	Comparator - Risk of Bias	Comparator - Applicability	Flow and Timing - Risk of Bias	Overall Risk of Bias
Alessio, G., et al.	2022	10.1128/spectrum.01858-22	Italy	High	Low	Unclear	Low	Not applicable	Not applicable	Low	High
de Maertelaere, E., et al	2020	10.1128/JCM.00159-20.	Belgium	Low	Low	Unclear	Low	Unclear	Low	Low	Moderate
Brantestig, S., et al	2020	https://doi.org/10.1111/apm.13025	Sweden	Low	Low	High	Low concern	High	Low	Low	High
Buron, V. and N. Banaei	2023	10.1128/jcm.00295-23	USA	Low	Low	Unclear	Low	Unclear	Low	Unclear	Moderate
Villalta, D., et al.	2020	10.1128/JCM.01436-20	Italy	Low	Low	Unclear	Low	Unclear	Low	Low	Moderate
Grassi, B., et al	2019	https://doi.org/10.1016/j.cca.2019.03.1158	Italy	Unclear	Unclear	Unclear	Low	Unclear	Low	Unclear	Moderate
Stojkovic, V., et al	2019	10.1016/j.cca.2019.03.1178	Belgium	High	Low	Unclear	Low	Unclear	Low	Unclear	High
Mehreen, A., et al.	2021		USA	High	Low	Unclear	Low	Unclear	Low	High	High
Altawallbeh, G., et al	2021	0.1093/jalm/jfab012	USA	Low	Low	Unclear	Low	Unclear	Low	Unclear	Moderate
Heireman, L., et al	2022	https://doi.org/10.1016/j.diagmicrobio.2021.115613	Belgium	Low	Low	Unclear	Low	Unclear	Low	Unclear	Moderate
Kadkhoda, K., et al.	2023	10.1128/spectrum.04754-22	USA	Low	Low	Unclear	Low	Unclear	Low	Low	Moderate
Fernández-Huerta, M., et al	2021	10.1128/JCM.00603-21	Spain	Low	Low	Unclear	Low	Unclear	Low	Low	Moderate
Bisognin, F., et al.	2020	https://doi.org/10.1128/JCM.00780-20.	Italy	Low	Low	Low	Low concern	Low	Low	Unclear	Moderate
Ruiz-Tagle, C., et al.	2024	10.1128/spectrum.00469-24	Chile	Low	Low	Low	Low concern	Low	Low	Low	Low
Cornaby, C., et al.	2022	10.1016/j.jim.2022.113340	USA	Low	Low	Unclear	Low	Not applicable	Not applicable	Moderate	Moderate
Khoury, R., et al	2023	https://doi.org/10.1093/clinchem/hvad097.250	USA	Low	Low	Unclear	Low	High	High	Unclear	High
