# Supplementary material for: Agreement and systematic bias between QuantiFERON chemiluminescent immunoassay and QuantiFERON enzyme-linked immunosorbent assay in the detection of latent tuberculosis infection: A systematic review and meta-analysis
Source: IJID Reg. 2025 Dec 7;18:100824. doi: 10.1016/j.ijregi.2025.100824 (PMC12809075; doi:10.1016/j.ijregi.2025.100824)
Supplement: Supplementary file 5 [file mmc5.pdf]

S3 Table: Certainty of evidence assessment using GRADE

Certainty assessment							№ of patients		Effect		Certainty	Importance
№ of studies	Study design	Risk of bias	Inconsistency	Indirectness	Imprecision	Other considerations	QFT-CLIA (LIASON)	QFT-ELISA	Relative (95% CI)	Absolute (95% CI)		
**Overall Agreement (%)**												
8	non-randomised studies	serious[a]	serious	not serious	not serious	none			not estimable		⨁◯◯◯ Very low[a]	IMPORTANT

CI: confidence interval

Explanations

a. Several studies had unclear or high risk of bias, particularly in the Index Test and Comparator domains. Only 2/8 studies were fully low risk across all domains.
